# Disseminated Nonsegmental Vitiligo Associated With Halo Nevi and Premature Gray Hair

**DOI:** 10.7759/cureus.13868

**Published:** 2021-03-13

**Authors:** Vignesh Ramachandran, Katelyn M Kim, Lisa Zhang

**Affiliations:** 1 Dermatology, Baylor College of Medicine, Houston, USA; 2 Dermatology, Henry Ford Health System, Detroit, USA

**Keywords:** halo nevi, vitiligo, melanoma, uveal melanoma, leukotrichia

## Abstract

Halo nevi (HN) are acquired melanocytic nevi circumferentially surrounded by a depigmented patch. HN are commonly associated with vitiligo and can be associated with uveal, mucosal, or cutaneous melanoma in certain patient populations. HN may also have localized leukotrichia of terminal hair overlying the nevus. We report a less common triad of rapid-onset HN, nonsegmental vitiligo, and premature hair graying (PHG) of scalp hair.

## Introduction

Halo nevi (HN; also known as Sutton's nevi) are acquired melanocytic nevi surrounded by a depigmented patch. HN may naturally resolve [1]. They are considered benign and affect one percent of Caucasians, appearing in childhood or early adulthood [2]. HN may be associated with nonsegmental vitiligo. We report a less common triad of rapid-onset HN, nonsegmental vitiligo and premature hair graying (PHG) of scalp hair.

## Case presentation

A healthy 20-year-old male presented to our clinic with a five-month history of asymptomatic depigmented lesions on his trunk and diffuse graying of his scalp hair. He noted an acute onset of these asymptomatic lesions slowly enlarging around nevi on his chest and abdomen. Three months later, he developed similar slowly enlarging areas of involvement disassociated from nevi on his back and progressive graying of his scalp hair only. He denied new or changing moles and reported no associated symptoms. Personal and family histories were non-contributory.

**Figure 1:**
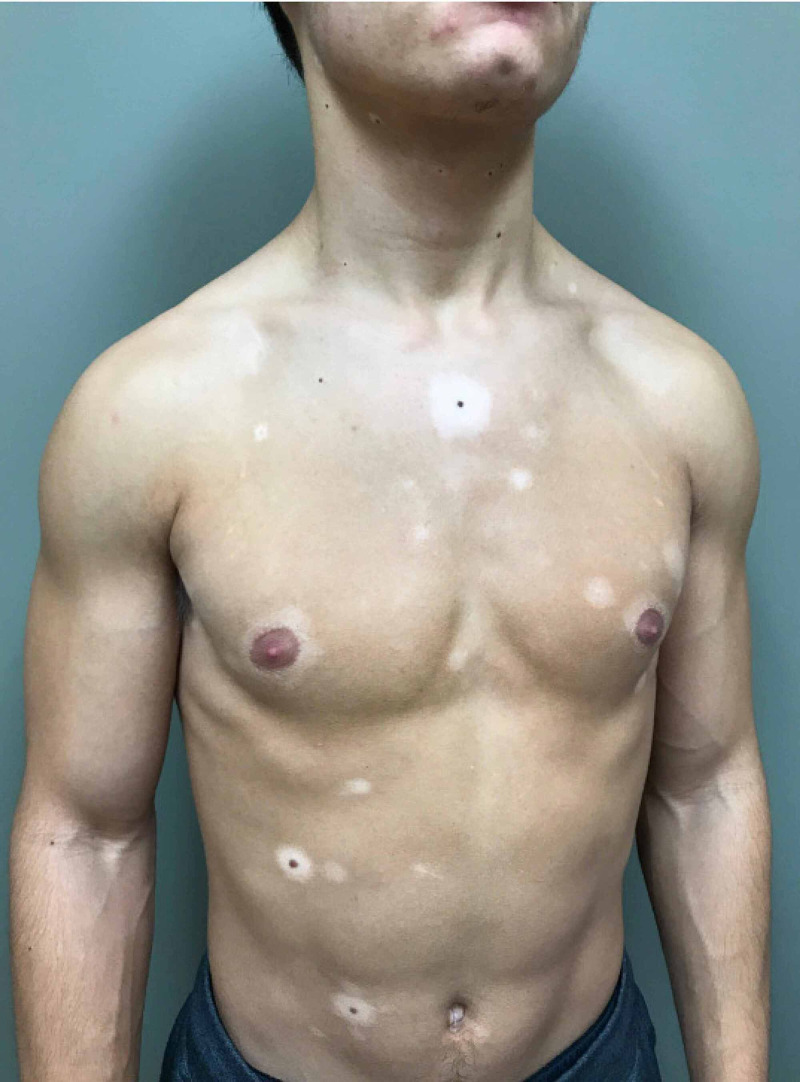
The torso and upper extremities of the patient revealing halo nevi and other lesions of vitiligo dissociated from nevi.

**Figure 2:**
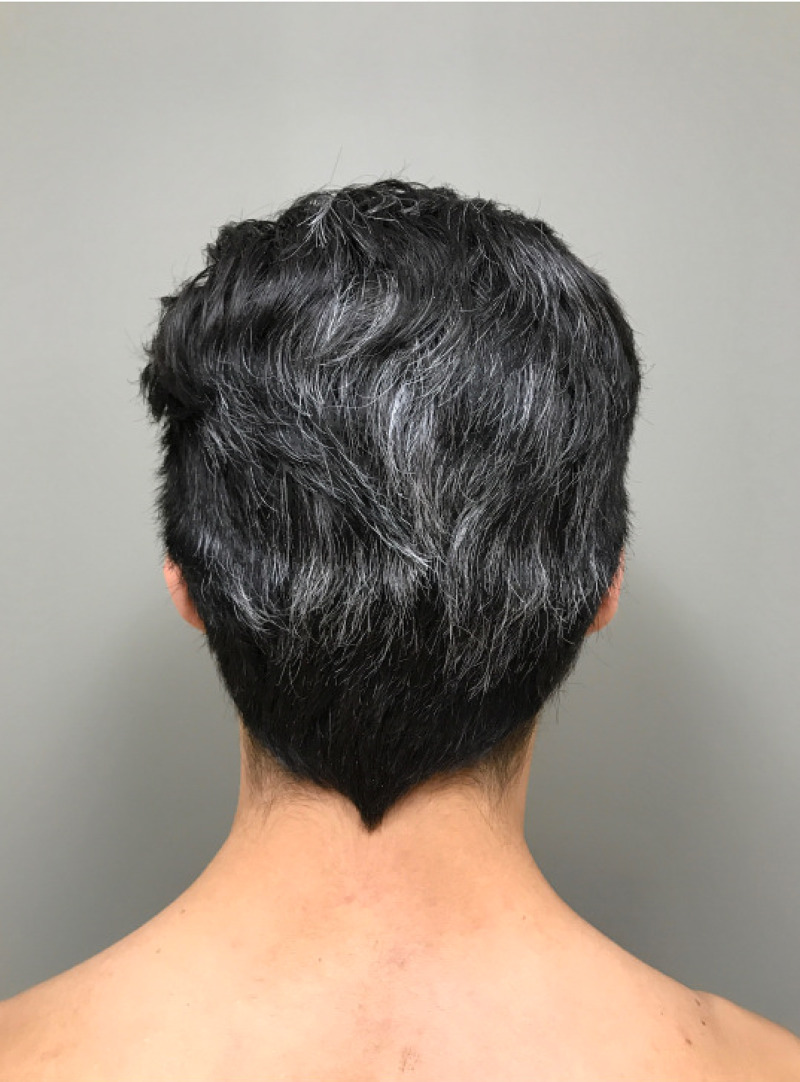
The patient’s hair notable for diffuse premature graying of hair.

On examination, he had numerous, isolated depigmented macules and patches on the trunk, with some surrounding symmetric, evenly pigmented 3- to 6-mm brown-black macules (Figure 1). Additionally, he had diffuse graying of his scalp hair without associated nevi (Figure 2). Overall, he had approximately five percent body surface area involvement. Wood’s lamp examination revealed enhancement of the depigmented patches. A diagnosis of acute, generalized vitiligo with associated PHG was made.

The patient was started on topical betamethasone dipropionate 0.05% ointment twice daily to the affected areas apart from the scalp. Gingko biloba (60 mg twice daily) and alpha-lipoic acid (100 mg once daily) capsules were also initiated. He was referred to ophthalmology and his ocular exam was unremarkable for melanoma. Thyroid-stimulating hormone was normal.

At four-month follow-up, the patient self-discontinued topical betamethasone dipropionate 0.05% ointment but continued to take gingko biloba and alpha-lipoic acid daily. He did not report new lesions or improvement to current depigmented spots. Due to insurance issues, he was unable to do narrowband ultraviolet light therapy or start topical tacrolimus ointment.

HN have two important associations: vitiligo and melanoma-associated leukoderma. The latter was ruled out with total body skin exam and ocular exam.

## Discussion

While the exact pathophysiology of HN is poorly understood, melanocytes are absent under histopathology similar to the histopathology of vitiligo, suggesting an etiologic link. HN are 8 to 10 times more common in vitiligo patients [3]. The inflammatory composition of HN is predominantly T-lymphocytes (4:1 ratio of CD8:CD4) with scattered macrophages, suggesting an unknown immunological mechanism of melanocyte destruction [4]. Overall, HN indicate an immune response targeting abnormal melanocytes - nevocytes [5]. This may indirectly relate to mechanisms implicated in vitiligo. Specifically, nonsegmental vitiligo is more often associated with other autoimmune diseases, such as autoimmune thyroiditis, than segmental vitiligo [1].

HN-associated vitiligo is more likely to present in certain populations and with certain clinical features. A retrospective study of 101 patients with HN-associated vitiligo demonstrated that risk factors for developing vitiligo in patients with HN include: 1) Koebner phenomenon, 2) multiple HN, and 3) family history of vitiligo [6]. A prospective study of 553 patients with HN-associated nonsegmental vitiligo (n=130) and generalized vitiligo (n=423) showed that age <18 years old, phototypes I-III, and trunk involvement were positively associated with HN-associated nonsegmental vitiligo. Involvement of the hands and feet were negative predictors of HN-associated nonsegmental vitiligo [1].

Our patient also had diffuse PHG. Lesional leukotrichia associated with vitiligo patches or HN of the scalp can result in focal graying of hair. However, diffuse PHG is uncommon. and family history of PHG may be seen in patients with nonsegmental HN-associated vitiligo [1,6]. However, reports of PHG occurring concurrently in a patient with an acute disseminated presentation of HN-associated vitiligo are lacking. PHG is an inherited trait; it is likely triggered by an early reduction in tyrosinase function in the melanocytes of the hair bulb and associated with defective migration of melanocytes in follicular papilla, and faulty melanocyte-cortical keratinocyte interactions [1]. However, genetic polymorphisms may suggest a possible immunological factor [7], targeting hair follicle melanocytes implicated in a subset of cases [1]. Nevertheless, PHG is a very unusual finding in a patient with HN-associated vitiligo. In patients with multiple HN, PHG may represent a robust immune response that warrants a search for melanoma as a possible trigger due to its association with multiple new HN [8]. Total body skin and ophthalmological exam were non-concerning in our patient.

Our patient’s acute clinical presentation of disseminated HN-associated nonsegmental vitiligo and diffuse PHG represents a rare presentation of vitiligo. While PHG may be related to an autoimmune/autoinflammatory process, it is not thought of as such. Thus, further research of the pathophysiology and clinical course of these findings in the context of HN-associated nonsegmental vitiligo may guide management and provide insights into disease prognosis.

## Conclusions

HN are benign melanocytic nevi with surrounding depigmented circular patches that can be associated with vitiligo, graying of the hair, and melanoma. Practitioners should be prompted to perform full skin examination or refer patients to dermatology, ophthalmology, or, if applicable, gynecology for internal examination to evaluate for cutaneous, mucosal, or uveal melanoma.

## References

[1] Ezzedine K, Diallo A, Léauté-Labrèze C (2012). Halo nevi association in nonsegmental vitiligo affects age at onset and depigmentation pattern. Arch Dermatol.

[2] Larsson PA, Lidén S (1980). Prevalence of skin diseases among adolescents 12-16 years of age. Acta Derm Venereol.

[3] Barona MI, Arrunátegui A, Falabella R, Alzate A (1995). An epidemiologic case-control study in a population with vitiligo. J Am Acad Dermatol.

[4] Patrizi A, Neri I, Sabattini E, Rizzoli L, Misciali C (2005). Unusual inflammatory and hyperkeratotic halo naevus in children. Br J Dermatol.

[5] Rodrigues M, Ezzedine K, Hamzavi I, Pandya AG, Harris JE; Vitiligo Working Group (2017). New discoveries in the pathogenesis and classification of vitiligo. J Am Acad Dermatol.

[6] Zhou H, Wu LC, Chen MK, Liao QM, Mao RX, Han JD (2017). Factors associated with development of vitiligo in patients with halo nevus. Chin Med J.

[7] Jin Y, Birlea SA, Fain PR (2010). Variant of TYR and autoimmunity susceptibility loci in generalized vitiligo. N Engl J Med.

[8] Naveh HP, Rao UNM, Butterfield LH (2013). Melanoma-associated leukoderma - immunology in black and white?. Pigment Cell Melanoma Res.

